# Lipidomics of HIV/HCV-related liver decompensation: association between plasma lipid depletion and immune dysregulation

**DOI:** 10.3389/fimmu.2026.1878207

**Published:** 2026-06-30

**Authors:** Raquel Behar-Lagares, Belen Requena, Juan Berenguer, Ana Virseda-Berdices, Juan Gónzalez-García, Carolina Gonzalez-Riano, Cristina Díez, Victor Hontañón, Aida Vaquero-Rey, Coral Barbas, Salvador Resino, Rubén Martín-Escolano, María Ángeles Jiménez-Sousa

**Affiliations:** 1 Unidad de Infección Viral e Inmunidad, Centro Nacional de Microbiología (CNM), Instituto de Salud Carlos III (ISCIII), Majadahonda, Madrid, Spain; 2 Centro de Metabolómica y Bioanálisis (CEMBIO), Facultad de Farmacia, Universidad San Pablo-CEU, CEU Universities, Urbanización Montepríncipe, Boadilla del Monte, Spain; 3 Centro de Investigación Biomédica en Red en Enfermedades Infecciosas (CIBERINFEC), Instituto de Salud Carlos III (ISCIII), Madrid, Spain; 4 Unidad de Enfermedades Infecciosas/VIH, Hospital General Universitario “Gregorio Marañón”, Madrid, Spain; 5 Instituto de Investigación Sanitaria Gregorio Marañón (IiSGM), Madrid, Spain; 6 Servicio de Medicina Interna-Unidad de VIH, Hospital Universitario La Paz, Madrid, Spain; 7 Instituto de Investigación Sanitaria La Paz (IdiPAZ), Madrid, Spain

**Keywords:** biomarkers, hepatitis C, HIV, lipidomics, liver decompensation

## Abstract

**Introduction:**

Globally, 15-30% of patients with chronic hepatitis C develop compensated advanced chronic liver disease (cACLD). cACLD may further advance to decompensated ACLD (dACLD), which is associated with higher liver-related mortality, even after successful antiviral treatment. This progression is accelerated by HIV coinfection, yet its underlying molecular mechanisms remain poorly understood.

**Methods:**

In this cross-sectional study, we characterized the plasma lipidomic profiles of 58 HIV/HCV-coinfected patients using untargeted liquid chromatography-mass spectrometry. Multivariate (OPLS-DA) and univariate (GLM) statistical models were employed to identify lipid species associated with disease severity.

**Results:**

We identified a signature of 28 lipids—predominantly phosphatidylcholines, phosphatidylethanolamines, and triglycerides— that were significantly depleted in patients with dACLD (17.2% of the cohort). This systemic lipid depletion showed relevant correlations with the pro-inflammatory chemokine IP-10 and soluble immune checkpoint proteins.

**Discussion:**

Our findings indicate that dACLD in HIV/HCV-coinfection is defined by a profound collapse in plasma lipids. This metabolic failure correlates with markers of inflammation and immune activation, suggesting that lipid dysregulation plays a critical role in the pathogenesis of liver decompensation.

## Graphical Abstract

**Figure:**
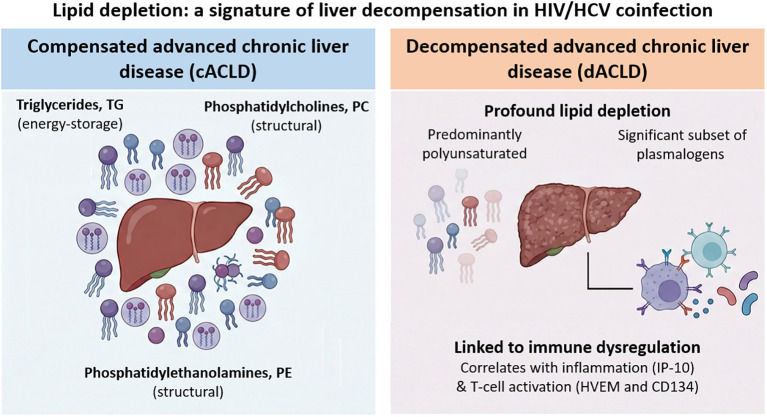

## 1 Introduction

Globally, a substantial proportion of individuals with chronic hepatitis C will progress to compensated advanced chronic liver disease (cACLD), a critical clinical stage defined by advanced fibrosis or cirrhosis (1). While often asymptomatic, cACLD places patients at high risk of transitioning to a decompensated state (dACLD), which is associated with a substantially increased risk of liver-related mortality (1). A central clinical challenge is that this risk persists even after achieving a virological cure for HCV, as the underlying drivers of liver injury often remain active (2). This issue is particularly exacerbated in the context of HIV coinfection, which is known to accelerate the progression to dACLD and its associated complications (3). Despite extensive characterization of the clinical predictors of liver decompensation, the underlying molecular mechanisms that trigger this shift remain poorly understood.

In advanced liver disease, the liver’s central role in lipid synthesis and transport becomes progressively impaired, leading to profound systemic lipid dysregulation (4, 5). These metabolic derangements may not simply reflect liver injury but could be linked to broader metabolic and immune disturbances. Lipid species, particularly glycerophospholipids (GP), are integral to cell membrane structure, and their alteration can compromise hepatocyte integrity and intracellular signalling (4, 6). Crucially, this lipid dysregulation is mechanistically linked to the immune dysfunction that characterizes advanced liver disease (7). Lipids can act as signalling molecules that directly modulate inflammatory pathways; for instance, changes in the lipid composition of T-cell membranes can impair their activation and contribute to the state of immune exhaustion frequently observed in chronic viral infections (7, 8).

Predicting the transition to dACLD remains clinically challenging, as it often occurs without clear preceding signs (1). Consequently, there is an urgent need for molecular biomarkers that reflect the underlying pathophysiology and can identify patients at the highest risk before irreversible deterioration occurs (2). Lipidomics has emerged as a powerful systems-level approach to capture these metabolic perturbations and their link to disease progression. Although prior metabolomic studies in HIV/HCV coinfection have reported associations between lipid−related pathways and advanced liver disease (9), to our knowledge none have specifically characterized the plasma lipidomic profile associated with liver decompensation in this high-risk population using a comprehensive untargeted lipidomics workflow.

This study aimed to characterize the plasma lipidomic profile that differentiates dACLD from cACLD in a high-risk cohort of patients with HIV/HCV-coinfection, and to investigate its links to systemic inflammation and immune dysregulation.

## 2 Materials and methods

### 2.1 Study design and participants

We conducted a cross-sectional study nested within the GeSIDA 10318 cohort (**Supplementary Data 1**). We included HIV/HCV-coinfected patients with advanced chronic liver disease (ACLD), defined as liver stiffness measurement (LSM) ≥10 kPa), obtained using transient elastography (FibroScan). Inclusion criteria required stable antiretroviral therapy (ART) for >6 months with undetectable HIV viral load and available frozen plasma samples. Exclusion criteria were hepatitis B coinfection, acute hepatitis C, or hepatocellular carcinoma.

### 2.2 Outcome variable

The outcome of interest was dACLD, defined by the presence of a Child-Turcotte-Pugh (CTP) score ≥7 or a history of clinical decompensation (ascites, bleeding esophageal varices, or hepatic encephalopathy). Patients not meeting these criteria were classified as compensated ACLD (cACLD). This combined definition integrates both functional impairment and clinical events, allowing a comprehensive characterization of advanced liver disease severity within the study cohort.

### 2.3 Non-targeted lipidomics analysis

Protocols regarding the reagents and standards, lipid extraction, quality management assurance, analytical conditions, lipid annotation, and data reprocessing and normalization can be found in **Supplementary Data 2**

### 2.4 Multiplex immunoassays and ELISA

Plasma markers were measured using Luminex 200™ technology (Luminex Corp., Austin, TX). We quantified inflammatory markers (IL-8, IL-18, IL-1RA, IP-10, MCP-1, TNF-RI) using ProcartaPlex™ assays. Additionally, soluble immune checkpoint proteins (ICPs) were measured using Immuno-Oncology Checkpoint Panels 1 and 3 (Invitrogen™), listed in **Supplementary Data 2**.

### 2.5 Statistical analysis

For the descriptive analysis, continuous variables were compared using the Mann-Whitney U test and categorical variables using the Chi-square test.

For lipidomic analysis, outliers were removed using the standard 1.5×IQR rule, and data were log-transformed (log10) and auto-scaled. Orthogonal partial least squares discriminant analysis (OPLS-DA) was performed to identify influential lipids (VIP >1), which was strictly used as an exploratory, heuristic feature-screening step to reduce dimensionality due to subgroup unbalance. The final 28-lipid signature was selected based on a sequential combination of criteria: only lipids with an OPLS-DA VIP score > 1.0 that subsequently achieved both statistical significance (adjusted GLM p-value < 0.05) and an exploratory false discovery rate threshold (FDR q-value < 0.20) in the covariate-adjusted GLMs were included. Significant lipids were subsequently determined using Generalized Linear Models (GLM) with a gamma distribution (log-link), adjusted for clinically relevant covariates such as age, gender, BMI, HCV viral load, and alcohol intake, previously selected by a stepwise method (forward), according to the specific model’s lowest Akaike information criterion (AIC). This stepwise selection was performed to identify the most parsimonious model for each lipid, preserving statistical power and preventing overfitting by adjusting only for covariates that genuinely contributed to model fit. P-values were corrected for multiple testing (False Discovery Rate (FDR)). Statistical significance was defined as a p-value<0.05 (two-tailed) and q-value <0.20, consistent with the exploratory nature of the analysis. An FDR threshold of 20% was chosen to balance the discovery of biologically meaningful lipidomic signatures with the control of false-positive rates in a discovery-phase clinical cohort, where tight physiological correlation among lipids can inflate false-negative rates under overly conservative corrections. This multi-step framework (OPLS-DA feature pre-screening, AIC-based covariate selection, and FDR correction) was specifically implemented to minimize multiple-testing inflation and reduce the risk of overfitting in the identification of the final lipid signature.

Associations between significant dACLD-related lipids and inflammatory biomarkers/immune checkpoint proteins (ICPs) were assessed using Spearman correlation. Correlations with r >0.25 or r <-0.25 were considered relevant. All correlation p-values were corrected for multiple testing using the FDR correction, and statistical relevance was defined as meeting both an unadjusted p-value < 0.05 and an FDR q-value < 0.20.

Analyses were performed using MetaboAnalyst 5.0 and R v4.3.1.

## 3 Results

### 3.1 Individuals’ characteristics

Baseline characteristics are detailed in **Table 1**. The cohort included 58 HIV/HCV-coinfected patients, of whom 10 (17.2%) had dACLD. Patients in the dACLD group had a history of decompensation events (ascites, variceal bleeding, or encephalopathy) and/or CTP ≥ 7, and also exhibited higher liver stiffness measurements. Other demographic and virological variables, including age, gender, and HCV genotype, were similar between groups. Biochemical markers are shown in **Supplementary Table 1**.

**Table 1:** Clinical, epidemiological, and virological characteristics of HIV/HCV-coinfected patients according to decompensated and compensated Advanced chronic liver disease.

Variable	All patients	dACLD	cACLD	*p*
**No.**	58	10 (17.2%)	48 (82.8%)	
Age (years)	51 (48–54)	53 (49–54)	51 (48–53)	0.635
Gender (male)	44 (79.5%)	8 (80.0%)	36 (75.0%)	0.999
BMI (kg/m^2^) (n =57)	24.4 (21.9–26.9)	24.3 (23.6–25.0)	24.4 (21.6–27.0)	0.906
Smoker (n = 56)				0.808
Never	6 (10.7%)	1 (11.1%)	5 (10.6%)	
Previous (>6 months)	14 (25.0%)	3 (33.3%)	11 (23.4%)	
Current	36 (64.3%)	5 (55.6%)	31 (66.0%)	
Alcohol intake (>50g/day) (n = 57)				0.071
Never	27 (47.4%)	2 (20.0%)	25 (53.2%)	
Previous (>6 months)	27 (47.4%)	8 (80.0%)	19 (40.4%)	
Current	3 (5.3%)	0 (0.0%)	3 (6.4%)	
Intravenous drug user				0.537
Never	13 (22.4%)	1 (10.0%)	12 (25.0%)	
Previous (>6 months)	45 (77.6%)	9 (90.0%)	36 (75.0%)	
Current	0 (0%)	0 (0%)	0 (0%)	
Previous HCV therapy	36 (62.1%)	7 (70.0%)	29 (60.4%)	0.834
Liver markers				
HSI (n = 56)	33.8 (29.3–37.2)	32.1 (29.9–37.1)	33.8 (29.2–37.2)	0.983
LSM (kPa)	26.0 (17.3–35.8)	34.7 (18.6–42.3)	25.7 (17.2–35.1)	0.308
APRI	1.9 (1.0–3.4)	1.3 (1.0–3.3)	1.9 (1.0–3.5)	0.536
TyG (n = 57)	8.7 (8.4–9.0)	8.6 (8.3–8.9)	8.7 (8.4–9.0)	0.796
TGHDL (n = 55)	2.8 (1.9–5.5)	2.8 (2.2–2.9)	2.8 (1.8–5.8)	0.838
METS-IR (n =53)	2.4 (2.3–2.7)	2.4 (2.4–2.5)	2.4 (2.3–2.7)	0.753
dACLD				
CTP≥7	1 (1.7%)	1 (10.0%)	0 (0.0%)	NA
Ascites (n = 57)	5 (8.8%)	5 (50.0%)	0 (0.0%)	
Bleeding esophageal varices	3 (5.2%)	3 (30.0%)	0 (0.0%)	
Hepatic encephalopathy	1 (1.7%)	1 (10.0%)	0 (0.0%)	
HCV markers				
HCV genotype				0.816
1	43 (74.1%)	7 (70.0%)	36 (75.0%)	
3	7 (12.1%)	1 (10.0%)	6 (12.5%)	
4	6 (10.3%)	1 (10.0%)	5 (10.4%)	
Others	2 (3.4%)	1 (10.0%)	1 (2.1%)	
Log_10_ HCV-RNA (IU/mL) (n = 57)	6.2 (5.7–6.7)	6.0 (5.2–6.1)	6.3 (5.8–6.7)	0.064
HCV-RNA > 850.000 IU/mL)	38 (65.5%)	5 (50.0%)	33 (68.8%)	0.442
HIV markers				
Previous AIDS (n = 57)	1 (1.8%)	0 (0.0%)	1 (2.1%)	0.999
Nadir CD4+/mm^3^ (n = 55)	135 (84–244)	158 (95–201)	130 (80–245)	0.735
Nadir < 200 CD4+/mm^3^ (n = 57)	39 (68.4%)	7 (70.0%)	32 (68.1%)	0.999
CD4+ T-cells/mm^3^	470 (303–700)	453 (334–604)	487 (314–714)	0.742
< 500 CD4+/mm^3^	31 (53.4%)	6 (60.0%)	25 (52.1%)	0.914
HIV antiretroviral therapy (n = 53)				0.137
NRTI + NNRTI	18 (34.0%)	0 (0.0%)	18 (40.9%)	
NRTI + II	24 (45.3%)	7 (77.8%)	17 (38.6%)	
NRTI + PI	6 (11.3%)	1 (11.1%)	5 (11.4%)	
Others	5 (9.4%)	1 (11.1%)	4 (9.1%)	

Statistics: The values are expressed as the absolute number (percentage) and median (interquartile range). P-values were calculated by the Chi-square test and the Mann-Whitney U test. dACLD, decompensated advanced chronic liver disease; cACLD, compensated advanced chronic liver disease; HSI, hepatic steatosis index; BMI, body mass index; HCV, hepatitis C virus; LSM, liver stiffness measurement; kPa, kilopascal; CTP, Child-Turcotte-Pugh score; APRI, AST to platelet ratio index; TyG, Triglycerides and glucose index; TGHDL, triglyceride/HDL-cholesterol ratio; METS-IR, Metabolic score for insulin resistance; NA, not applicable; HCV-RNA, viral load of hepatitis C; AIDS, acquired immune deficiency syndrome; NRTI, nucleoside analogue HIV reverse transcriptase inhibitor; NNRTI, non-nucleoside analogue HIV reverse transcriptase inhibitor; II, HIV integrase inhibitor; PI, HIV protease inhibitor.

### 3.2 Lipidomic signature of liver decompensation

**Figure 1:**
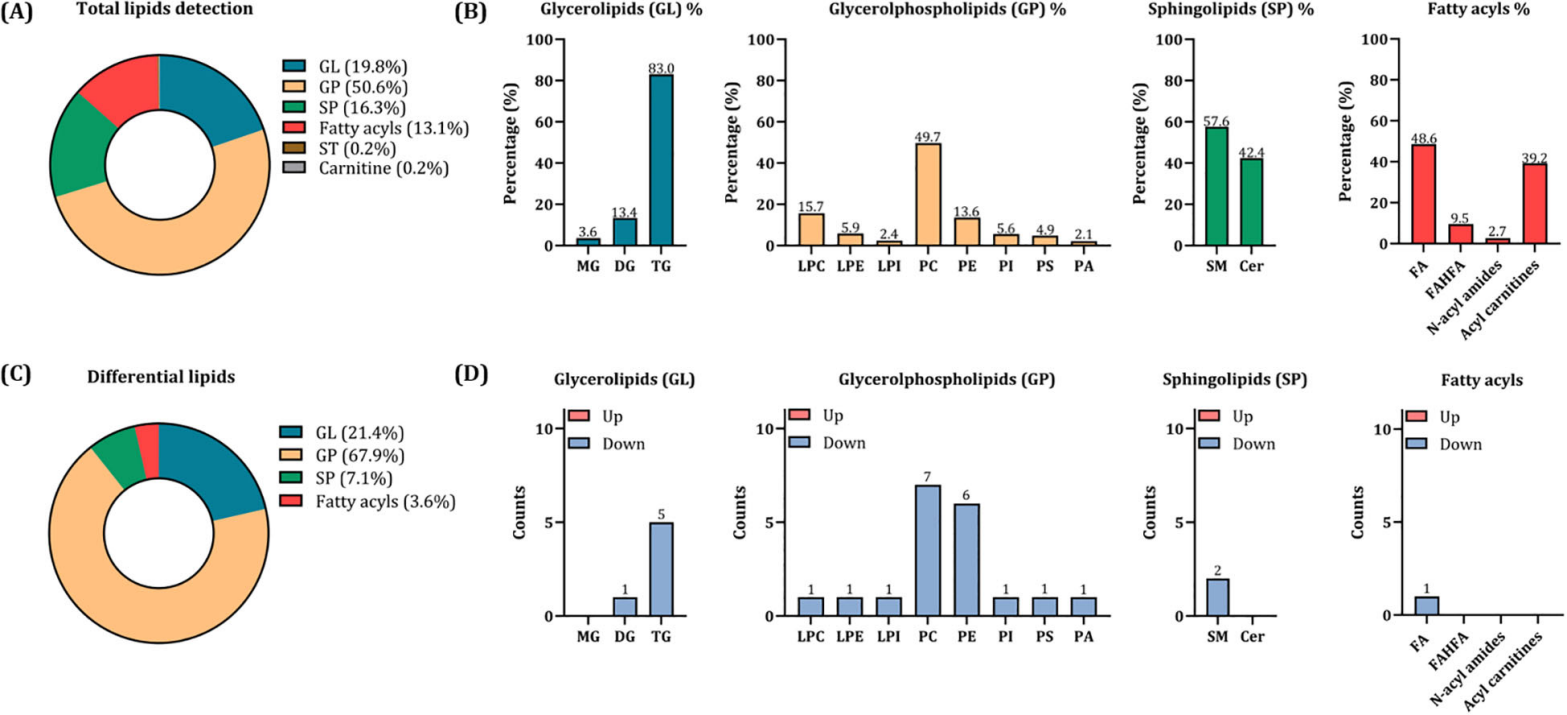
Lipidomic analysis in patients with HIV/HCV according to decompensated advanced chronic liver disease (dACLD). **(A)** Proportion of main classes of identified lipids. **(B)** Proportion of lipid subclasses in each main class. **(C)** Proportion of main classes of differential lipids according to dACLD. **(D)** Counts of up- or down-regulated differential lipids in dACLD vs cACLD patients with HIV/HCV. Glycerolipids (GL) are referred to monoglycerides (MG), diglycerides (DG) and triglyceride (TG); glycerolphospholipids (GP) to lysophosphatidylcholines (LPC), lysophosphatidylethanolamines (LPE), lysophosphatidylinositols (LPI), phosphatidylcholines (PC), phosphatidylethanolamines (PE), phosphatidylinositols (PI), phosphatidylserines (PS) and phosphatidic acids (PA); sphingolipids (SP) to sphingomyelins (SM) and ceramides (Cer); and fatty acyls to fatty acids (FA), fatty acyl ester of hydroxy fatty acid (FAHFA), N-acyl amides and Acyl carnitines.

We identified 566 distinct plasma lipid species, classified according to the LIPID MAPS structure database (LMSD) (**Figures 1A, B**).

Multivariate OPLS-DA models were utilized to explore the separation between dACLD and cACLD patients (**Supplementary Figure 1A, B**). For the positive ionization mode (LC-MS ESI+), the model exhibited a cumulative R^2^Yof 0.734 (p = 0.216 under 1, 000 permutations) and a predictive Q^2^ of −0.458 (p = 0.690 under 1, 000 permutations) (**Supplementary Figure 1C**). Similarly, the negative ionization mode (LC-MS ESI-) model showed a cumulative R^2^Y of 0.423 (p = 0.374 under 1, 000 permutations) and a predictive Q^2^ of −0.274 (p = 0.491 under 1, 000 permutations) (**Supplementary Figure 1D**). These parameters (negative Q2 values and non-significant permutation p-values) indicate global multivariate model overfitting due to subgroup unbalance (n=10 dACLD vs. n=48 cACLD); therefore, the OPLS-DA was strictly employed as a heuristic feature-screening step to select lipids with a VIP score > 1.0. Subsequently, adjusted GLM analysis of these VIP-filtered lipids identified a specific signature of 28 lipids significantly associated with dACLD. Notably, all 28 lipids were depleted in patients with dACLD compared to cACLD (**Figure 1C, D**; **Supplementary Table 2**).

**Figure 2:**
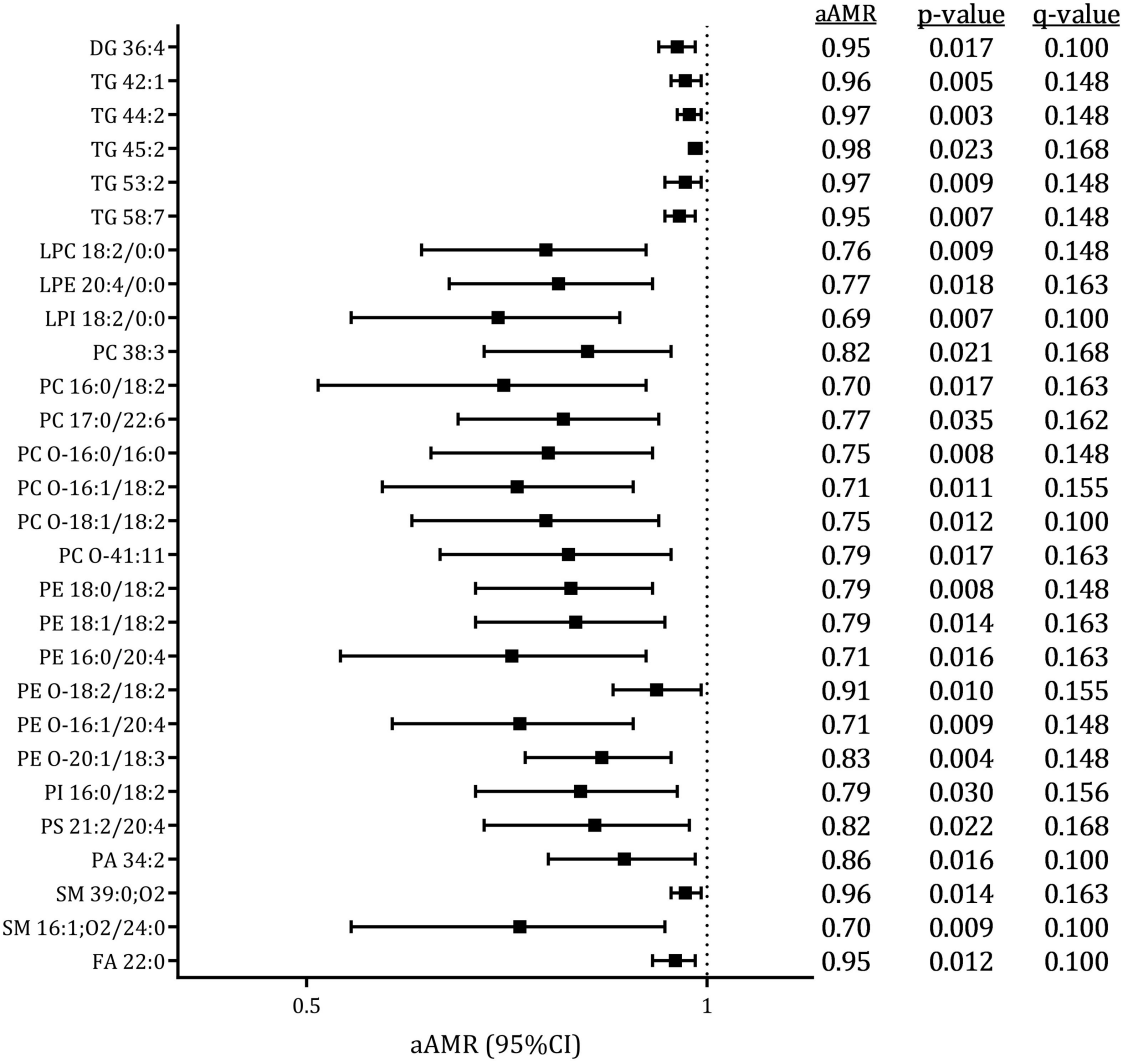
Plasma lipids associated with decompensated advanced chronic liver disease (dACLD) in patients with HIV/HCV. Statistics: Data were calculated by Generalized Linear Models (GLM) with a gamma distribution (log-link), adjusted by age, gender, body mass index (BMI), HCV viral load, and alcohol intake, previously selected by a stepwise method (forward). The q-values represent p-values corrected for multiple testing using the False Discovery Rate (FDR). aAMR, adjusted arithmetic mean ratio; DG, diglyceride; TG, triglyceride; LPC, lysophosphatidylcholine; LPE, lysophosphatidylethanolamine; LPI, lysophosphatidylinositol; PC, phosphatidylcholine; PC O, plasmalogen PC; PE, phosphatidylethanolamine; PE O, plasmalogen PE; PI, phosphatidylinositol; PS; phosphatidylserine; PA, phosphatidic acid; SM, sphingomyelin; FA, fatty acid.

This signature was dominated by glycerophospholipids (67.9%), particularly phosphatidylcholines (PC) and phosphatidylethanolamines (PE), followed by glycerolipids (21.4%), primarily triglycerides (TG). Structurally, these depleted lipids were exclusively long-chain species, predominantly polyunsaturated (82.1%), and included a substantial subset of plasmalogens (**Figure 2**).

### 3.3 Correlation between significant lipids and immune biomarkers

**Figure 3:**
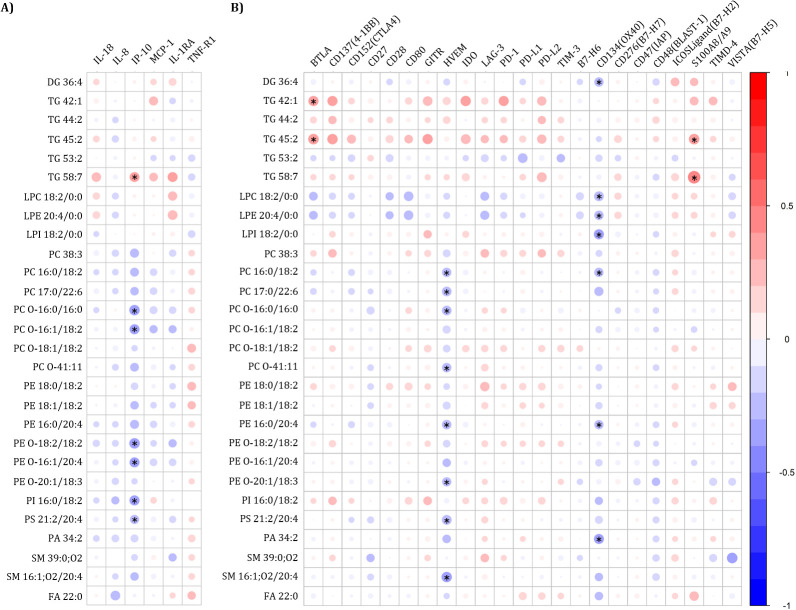
Spearman correlation plot between significant plasma lipids and plasma **(A)** inflammatory-related biomarkers and **(B)** immune checkpoint proteins (ICPs) across the entire study cohort (N = 58). The size of the circles is proportional to the strength of the correlation. The color represents the direction (colour legends are shown on the right), whereas large dark red and blue represent strong positive and negative correlations, respectively. Lipids are on the vertical axis, and inflammatory-related biomarkers and immune checkpoint proteins are on the horizontal axis. Those correlations with rho>0.25 o rho<-0.25, p-value<0.05, and q-value<0.2 are shown with an asterisk. DG, diglyceride; TG, triglyceride; LPC, lysophosphatidylcholine; LPE, lysophosphatidylethanolamine; LPI, lysophosphatidylinositol; PC, phosphatidylcholine; PC O, plasmalogen PC; PE, phosphatidylethanolamine; PE O, plasmalogen PE; PI, phosphatidylinositol; PS; phosphatidylserine; PA, phosphatidic acid; SM, sphingomyelin; FA, fatty acid; IL, interleukin; IP-10, human interferon-inducible protein 10; MCP-1, monocyte chemoattractant protein-1; IL-1RA, IL-1 receptor antagonist; TNF-RI, tumor necrosis factor receptor-1; BTLA, B, and T lymphocyte attenuator; CD, cluster of differentiation; GITR, glucocorticoid-induced TNFR-related; HVEM, herpesvirus entry mediator; IDO, indoleamine 2, 3-dioxygenase; LAG-3, lymphocyte activation gene-3; PD-1, programmed cell death protein 1; PD-L1, programmed death-ligand 1; PD-L2, programmed death-ligand 2; TIM-3, T-cell immunoglobulin and mucin-domain containing-3; TIMD-4, T-cell immunoglobulin and mucin domain-containing protein 4; VISTA(B7-H5), V-domain Ig suppressor of T cell activation.

Correlation analysis across the entire cohort (N = 58) revealed distinct associations between the lipidomic signature and immune markers (**Figure 3**). Depleted structural lipids (PC and PE) showed widespread negative correlations with the pro-inflammatory chemokine IP-10 and the immune checkpoints HVEM and CD134. In contrast, specific depleted TG species (e.g., TG 42:1, TG 58:7) exhibited positive correlations with the inhibitory checkpoint BTLA and the inflammatory marker S100A8/A9.

## 4 Discussion

This study characterizes the plasma lipidomic profile associated with dACLD in patients with HIV/HCV-coinfection. Our findings reveal a distinct plasma lipid profile in dACLD, primarily defined by a significant reduction of GP and GL. Furthermore, the consistent correlations observed between these depleted lipids and key markers of inflammation and immune dysregulation, point to a close interplay between impaired lipid metabolism and the systemic immune activation that fuels the advancement of liver disease. This specific signature of lipid depletion is likely driven by the complex interplay between HIV/HCV coinfection and progressive loss of hepatic reserve.

The significant depletion of PC and PE observed in dACLD patients highlights a critical disruption in hepatic phospholipid homeostasis. Reduced plasma PC levels are consistent with previous reports linking lipid alterations to fibrosis progression (4, 10) and likely reflect the impaired hepatic synthesis and secretion of lipoproteins characteristic of advanced liver failure (6). Similarly, the decrease in PE could reflect potential alterations consistent with mitochondrial dysfunction, given PE’s essential role in maintaining mitochondrial inner membrane structure and the electron transport chain (11). Since disrupted PE homeostasis has been previously linked to ER stress and apoptosis (5), this combined depletion of structural lipids may point to an altered hepatocyte integrity potentially contributing to the severity of liver decompensation.

Regarding GL, while routine clinical measurements (total triglycerides and cholesterol) failed to distinguish between cACLD and dACLD, lipidomic profiling revealed a specific and profound depletion of long-chain TG in the decompensated group. This discrepancy underscores the limitations of standard biochemical panels, which only measure total lipid pools and lack the molecular resolution to detect the selective remodeling of lipid species. Similarly, conventional functional markers (such as glucose, albumin, or bilirubin) often remain homeostatically buffered or clinically managed, failing to reflect early subcellular perturbations. In contrast, untargeted lipidomics bypasses these limitations by directly capturing early, specific alterations, which signal systemic metabolic collapse before standard clinical markers deviate significantly. This systemic hypolipidemia likely reflects the progressive failure of hepatic very-low-density lipoproteins (VLDL) synthesis and secretion, a characteristic feature of advanced liver disease (6, 12). Mechanistically, impaired VLDL assembly—dependent on apolipoprotein B and microsomal triglyceride transfer protein (MTTP)—leads to a paradox where intracellular TG retention coexists with low plasma TG levels (13). In the context of chronic hepatitis C, this reduction in serum VLDL-TG fractions parallels fibrosis progression and diminished hepatic export capacity (14). Consequently, the observed depletion of long−chain TG could reflect disrupted hepatic lipid handling in advanced liver disease.

A detailed examination of the 28 depleted lipid species reveals specific structural characteristics that provide deeper insight into the pathophysiology of dACLD. Notably, these lipids were predominantly polyunsaturated (PUFAs) and included a significant subset of plasmalogens. The selective depletion of these long-chain polyunsaturated species reflects specific pathological vulnerabilities. First, the high density of double bonds in PUFAs renders them highly susceptible to lipid peroxidation under the heightened oxidative stress conditions that characterize HIV/HCV coinfection and hepatic decompensation (15), leading to their preferential degradation. Second, the synthesis of long-chain PUFAs is highly dependent on liver-specific elongases and desaturases, whose activity is markedly impaired during the progressive loss of functional hepatocyte volume in dACLD (8). Third, the selective impairment of hepatic lipoprotein packaging (VLDL) prevents the systemic distribution of these newly synthesized PUFA-containing lipids (14). Together, these mechanisms explain why the loss of these complex long-chain species serves as a highly sensitive biomarker of systemic metabolic failure, whereas standard cumulative lipid measurements remain unaltered.

This selective depletion suggests a dual pathological impact. First, the loss of PUFAs implies a structural deficit leading to increased membrane rigidity and a compromised capacity to generate anti-inflammatory lipid mediators (16). Second, the reduction in plasmalogens—key endogenous antioxidants—strongly indicates a state of heightened oxidative stress where protective lipids are consumed faster than they are synthesized (17). This loss of antioxidant defense likely accelerates liver damage, contributing to mitochondrial dysfunction and cell death. Collectively, this generalized loss of long-chain lipids, which are primarily synthesized and packaged by the liver, underscores the progression from a localized hepatic pathology to a systemic metabolic collapse, reflecting the liver’s inability to supply the body with the essential structural and energetic building blocks required for systemic homeostasis (6).

Our findings suggest these metabolic derangements are intricately linked to the immune dysregulation characteristic of advanced liver disease. Notably, the correlational patterns differ markedly between structural (PC and PE) and energy-storage lipids (TG), revealing distinct and potentially complementary roles in the metabolic-immune interactions that contribute to liver decompensation.

Structural phospholipids, such as PC and PE, were consistently and strongly negatively associated with the pro-inflammatory chemokine IP-10 and the co-stimulatory ICPs HVEM and CD134. IP-10 is a well-known marker of inflammation and liver injury (18), often elevated in chronic HCV and HIV infections (19), while HVEM and CD134 are critical for T-cell activation (20). This inverse relationship suggests that as the liver’s capacity to maintain phospholipid homeostasis declines, a pro-inflammatory and immune-activated state intensifies. The depletion of these structural lipids may disrupt cell membrane integrity and signaling, potentially exacerbating immune dysregulation and contributing to T-cell exhaustion, factors that could play a role in accelerating disease progression. This observation is consistent with established evidence that lipid dysregulation contributes to hepatic inflammation and oxidative stress (4, 5).

In contrast, energy-storage TG lipids showed positive correlations with the inhibitory checkpoint BTLA and the inflammatory marker S100A8/A9. BTLA suppresses T-cell activation (21), while S100A8/A9 modulates inflammatory responses (22). In the context of dACLD, where circulating TG are low due to hepatic failure, these positive correlations may indicate that this profound metabolic collapse is linked to a dysfunctional immune state unable to mount effective responses (7). These findings highlight that different lipid classes are associated with divergent immune pathways that collectively contribute to liver decompensation.

Regarding clinical translation, while untargeted LC-MS serves as a powerful discovery-phase tool, its high complexity and cost currently limit its direct bedside application. However, for clinical implementation, this 28-lipid signature, or a reduced subset of these biomarkers, could be adapted into targeted LC-MS/MS assays, which are more suitable for routine clinical workflows. Although further validation and standardization are required, such approaches may facilitate early risk stratification and preventive management of patients at high risk of decompensation, potentially reducing the burden of advanced liver disease.

Our study is limited by its cross-sectional design, which precludes causal inference. Additionally, although the sample size was statistically adequate for metabolomic analysis based on MetSizeR, these findings require validation in larger, independent cohorts, as the relatively small number of patients with dACLD underscores their exploratory nature. We also acknowledge that our global multivariate OPLS-DA models exhibited overfitting (reflected by negative Q2 values and non-significant permutation tests), which is a common limitation in heterogeneous clinical cohorts with unbalanced subgroups. To mitigate this multidimensional overfitting, OPLS-DA was strictly employed as an initial heuristic screening tool to reduce dataset dimensionality (VIP > 1.0). Crucially, the final 28-lipid signature was determined and validated using covariate-adjusted univariate GLMs coupled with Benjamini-Hochberg FDR correction, supporting the robustness of the identified association while accounting for key clinical confounders. Nevertheless, given the exploratory nature of this study, both the identified 28-lipid signature and its correlation patterns with immune biomarkers must be interpreted with caution as hypothesis-generating trends that require validation in larger, independent prospective cohorts before any clinical translation.

In conclusion, we identified a profound depletion of plasma GP and GL as a lipid signature associated with ACLD in HIV/HCV coinfection. This metabolic pattern correlates with systemic inflammation and immune checkpoint activation, suggesting a critical interplay between lipid dysregulation and viral-related immune dysregulation. These lipids represent potential exploratory biomarkers associated with liver decompensation that require prospective validation in larger, independent cohorts before clinical implementation.

## Data Availability

The raw data supporting the conclusions of this article will be made available by the authors, without undue reservation. Requests to access these datasets should be directed to jimenezsousa@isciii.es.

## Supplementary material

Supplementary Data Sheet 1 Members of the GeSIDA 10318 Study Group.

Supplementary Data Sheet 2 Additional description of methods section.

Supplementary Table 1 Biochemical markers of patients with HIV/HCV according to decompensated and compensated Advanced chronic liver disease.

Supplementary Table 2 Significant associations of lipids with decompensated advanced chronic liver disease (dACLD) in patients with HIV/HCV.
