# Supplementary material for: Lipidomics of HIV/HCV-related liver decompensation: association between plasma lipid depletion and immune dysregulation
Source: Front Immunol. 2026 Jun 30;17:1878207. doi: 10.3389/fimmu.2026.1878207 (PMC13364979; doi:10.3389/fimmu.2026.1878207)
Supplement: Supplementary Data Sheet 1 — Members of the GeSIDA 10318 Study Group. [file DataSheet1.docx]

Supplementary Material

# Supplementary Data 1. Members of the GeSIDA 10318 Study Group.

Hospital General Universitario Gregorio Marañón, Madrid: A Carrero, P Miralles, JC López, F Parras, B Padilla, T Aldamiz-Echevarría, F Tejerina, C Díez, L Pérez-Latorre, C Fanciulli, I Gutiérrez, M Ramírez, S Carretero, JM Bellón, J Bermejo, and J Berenguer.

Hospital Universitario La Paz, Madrid: V Hontañón, JR Arribas, ML Montes, I Bernardino, JF Pascual, F Zamora, JM Peña, F Arnalich, M Díaz, J González-García.

Hospital Universitari Vall d'Hebron, Barcelona: E Van den Eynde, M Pérez, E Ribera, M Crespo.

Hospital Universitario Príncipe de Asturias, Alcalá de Henares: A Arranz, E Casas, J de Miguel, S Schroeder, J Sanz.

Hospital Donostia, San Sebastián: MJ Bustinduy, JA Iribarren, F Rodríguez-Arrondo, MA Von-Wichmann.

Hospital Universitario de La Princesa, Madrid: J Sanz, I Santos.

Hospital Clínico San Carlos, Madrid: J Vergas, MJ Téllez.

Hospital Clínico Universitario, Valencia: A Ferrer, MJ Galindo.

Hospital Universitario Ramón y Cajal, Madrid: JL Casado, F Dronda, A Moreno, MJ Pérez-Elías, MA Sanfrutos, S Moreno, C Quereda.

Hospital General Universitario, Valencia: L Ortiz, E Ortega.

Fundación SEIMC-GESIDA, Madrid: M Yllescas, P Crespo, E Aznar, H Esteban

# Supplementary Data 2. Additional description of methods section.

**Viral inactivation of plasma samples**

Viral inactivation of plasma samples was performed by mixing 300 μL of plasma with 900 μL of cold MeOH:EtOH (1:1, v/v), reaching a 1:3, v/v ratio. After that, the samples were vortex mixed for 1 min and introduced on an ice-bath for 5 min. Finally, the samples were centrifuged at 16000g, during 20 min and 4ºC, and then they were stored at -80ºC until analysis.

**Non-targeted lipidomics analysis**

**Reagents and standards**

LC-MS grade methanol (MeOH), acetonitrile (ACN), and isopropanol (IPA) were obtained from Fisher Scientific (Pennsylvania, United States). Analytical grade ammonia solution (28%, GPR RECTAPUR®) and acetic acid glacial (AnalaR® NORMAPUR®) were obtained from VWR Chemicals (Pennsylvania, United States). The ammonium fluoride (NH4F) (ACS reagent, ≥ 98%) and Methyl-tert‑butyl ether (MTBE) were purchased from Sigma‐Aldrich (Steinheim, Germany). Reverse‐osmosed ultrapure water for aqueous solutions was obtained from a Milli‐Qplus185 system (Millipore, Billerica, MA, USA). For analytical quality assurance, C17–Sphinganine and d31–palmitic acid were purchased from Avanti Polar Lipids (Alabaster, Alabama, USA) for LC-MS. In the same way, for CE-MS quality assurance, methionine sulfone, paracetamol, 2-(N-morpholino)ethanesulfonic acid (MES), and formic acid were acquired from Sigma (Steinheim, Germany). Reference mass solutions for LC-MS and CE-MS were obtained from Agilent Technologies.

**Lipid extraction**

Inactivated plasma samples were processed at the "Centro de Metabolómica y Bioanálisis, CEMBIO" (Madrid, Spain), following an extraction method consisting of MeOH:MTBE (1:1, v/v), which was tested and developed in our laboratory. Briefly, the samples were thawed on ice and vortex-mixed for 2 minutes. On the ice, we pipetted 200 μL of sample, consisting of 50 µL of plasma and 150 µL of MeOH:EtOH (1:1, v/v), to an Eppendorf tube. Then, we added 175 μL of MTBE (RT) and vortex-mixed samples for 30 min. In addition, a mixture of nonendogenous internal standards (1 ppm of C17-Sphinganine and 2 ppm d31-palmitic acid) was added to each sample. Following, the samples were centrifuged for 15 minutes at 16.100xg and 15⁰C. We transferred 100 μL of the supernatant into clean HPLC-MS (Thermo Fisher Scientific, Madrid, Spain) vials with glass insert and centrifuged at 2000g and 15⁰C for 5 min before injecting into the system.

**Quality management assurance and blank samples**

Quality Control (QC) samples were prepared by polling equal volumes of each plasma sample, 30 μL in our case, to an Eppendorf tube. These QC samples were then processed in parallel with the rest of the experimental samples in the same manner. During the run, the system's stability, performance, and sample treatment method repeatability were all monitored. Along with the other samples, two blank samples were prepared using the same lipid extraction procedure with the sample solvents and were injected at the beginning and end of the analytical sequence to find common contaminations.

**Analytical conditions selected for a lipidomics analysis**

We performed an untargeted lipidomics analysis to cover the broader spectrum of plasma lipidome. Samples were analyzed using an Agilent 1290 Infinity II Ultra-High-Performance Liquid-Chromatography (UHPLC) system coupled to an Agilent 6546 Quadrupole Time-of-Flight (QTOF) Mass Spectrometer (MS) equipped with dual Agilent Jet Stream (AJS) Electrospray (ESI) ion source. The Agilent 1290 Infinity II Multisampler system was used to uptake 1 μL in ESI(+) and ESI(-) of the extracted plasma samples, set at a temperature of 15 °C to avoid lipid precipitation. An Agilent InfinityLab Poroshell 120 EC – C18 (3.0 ×100 mm, 2.7 μm) (Agilent Technologies) reverse phase column and compatible guard column (Agilent InfinityLab Poroshell 120 EC –C18, 3.0 ×5 mm, 2.7 μm) was used and held at 50 °C 1.

The Agilent 6546 QTOF mass spectrometer, equipped with a dual AJS ESI ion source, setting parameters were as follows: 150 V fragmentor, 65 V skimmer, 3500 V capillary voltage, 750 V octopole radio frequency voltage, 10 L/min nebulizer gas flow, 200 °C gas temperature, 50 psi nebulizer gas pressure, 12 L/min sheath gas flow, and 300 °C sheath gas temperature. Data were collected in separate runs in positive and negative ESI modes, operated in full scan mode from 40 to 1700 m/z with a scan rate of 3 spectra/s. A solution consisting of two reference mass compounds was used throughout the whole analysis: purine (C5H4N4) at m/z 121.0509 for ESI(+) and m/z 119.0363 for ESI(-); and HP-0921 (C18H18O6N3P3F24) at m/z 922.0098 for ESI(+) and m/z 980.0163 (HP-0921 + acetate) for ESI(-). An Agilent 1260 Iso Pump was used to continuously infuse the masses into the system at a 1 mL/min (split ratio 1:100) to provide a constant mass correction 1.

The mobile phases used for both positive and negative ionization modes consisted of (A) 10 mM ammonium acetate and 0.2 mM NH4F in H2O/MeOH (9:1, v/v) and (B) 10 mM ammonium acetate, 0.2 mM NH4F in ACN/MeOH/IPA (2:3:5, v/v/v). A solvent mixture of MeOH:IPA (50:50, v/v) was used for the multiwash strategy, with a wash time set at 15 s and an A/B (30:70, v/v) mixture to assist in the starting conditions. The chromatography gradient started at 70% of B at 0 –1 min, 86% B at 3.5 –10 min, and 100% B at 11–17 min. Starting conditions were recovered by min 17, followed by a 2 min re-equilibration time, reaching a total running time of 19 min. The flow rate was kept constant at 0.6 mL/min during the analysis.

At the end of the analysis, iterative MS/MS acquisition mode was performed for both ionization modes using QC samples. Two different collision energies, 20 eV and 40 eV, were used to conduct ten measurements (five measurements at each one), and they were operated with an MS and MS/MS scan rate of 3 spectra/s, 3 precursors per cycle, a mass range of m/z 40 - 1700, a narrow (~ 1.3 amu) MS/MS isolation width, and 5000 counts and 0.001 % of MS/MS threshold. The software chooses the three more intense precursor ions for subsequent fragmentation to obtain their MS/MS spectra in each measurement. In the second measurement of the same sample, the software discards the first three previously selected precursor ions and selects the next three more intense precursor ions at the same precise time point. In addition, reference masses and contaminants detected in blank samples were excluded, preventing their inclusion in the iterative MS/MS runs. By analyzing the sample many times, we were able to obtain the MS/MS information of most of the plasma lipidome by collecting thousands of MS/MS spectra using MassHunter Workstation Software LC-MS Data Acquisition v B.09.00 (Agilent Technologies, Waldrobonn, Germany).

**Lipid annotation process**

The lipid Annotation process was performed following a lipid annotation strategy developed by our laboratory 2. This strategy consisted of the use of a combination of bioinformatics tools based on three different annotation mechanisms: spectra matching (Lipid Annotator, MS-Dial), the bottom-up strategy (LipidHunter) and the fragment intensity rules (LipidMS). Following this approach, we significantly improved the lipid annotation process. The software parameters were set as follows.

Lipid Annotator v1.0 (Agilent) 3: Q-Score ≥ 20.0, adduct selection H+, Na+ and NH4+ for positive ion mode and H- and C2H3O2- for negative ion mode. We selected all lipid classes to perform an untargeted analysis. For the ID parameters, the Mass Threshold was set at mass deviation ≤ 20.0 ppm, the “Report top candidate only” option was selected, the Fragment score was ≥ 30, the Total score was ≥ 60, and the Constituent Level was ≥ 10%.

MS-DIAL 4 (Riken) 4: the raw data files were converted into “.ibf” files using the MS IBF file Converter software. For MS/MS data reprocessing, we selected the soft ionization for LC-MS/MS, chromatography separation type, conventional LC-MS method type, and profile data as the data type. We selected the corresponding ionization mode (positive or negative) for each analysis and, Lipidomics was used as the target omics. The MS1 and MS/MS m/z detection window was set at 40 – 1700 Da, and the retention time window was set at 0 – 19 minutes. The smoothing level for the peak detection window was set as 1 scan. The Accurate mass tolerance was 0.01 Da for MS1 and 0.025 for MS2 for the Identification window, and the identification score cut-off was set at 70%. Next, we selected specific adducts depending on the ionization mode we analyzed (H+, Na+ and NH4+ for positive and H- and C2H3O2- for negative ion mode). The rest of the parameters were set as default.

LipidHunter 5: we set from 0 – 19 min the scan range, 40 – 1700 m/z range, +/- 0.75 m/z precursor window, DDA Top 6, +/- 20 ppm for MS tolerance level, the absolute intensity for the MS level threshold was set at 1000, +/- 20 ppm MS/MS tolerance level, the absolute intensity for the MS/MS level threshold was set at 10, 80% isotope score, 75% Rank score and 0.10% as the minimum relative intensity for the scoring.

LipidMS 3.0 6, 7: We reprocessed our data by selecting the Batch processing option in the corresponding ionization mode. The m/z tolerance for MS1 and MS/MS was set at 20 ppm. The tolerance for the RT window was set at 30 seconds. The rest of the parameters were set as default. Finally, the lipid classes selected for the annotation process were established according to the ionization mode.

Subsequently, redundant data and false positive annotations were eliminated by combining and manually curating the collected information. In addition, the tentative annotation provided by the software annotation tools was combined with the manual inspection of the MS and MS/MS spectra data of the samples, based on the fundaments of structural elucidation and the assistance of the CEU Mass Mediator (CMM) free access technology 8 to corroborate the accuracy of the lipid annotations.

**Data reprocessing**

The raw data files were reprocessed by performing the hybrid strategy 2, which consisted of using an extensive lipid compound database, which was imported into the Agilent MassHunter Profinder software (B.10.0.2, Agilent Technologies, Santa Clara, CA, USA) to perform the time alignment and feature extraction using the "Batch Targeted Feature Extraction" mode. This strategy allowed us to ascertain the differences in the plasma lipidomic fingerprinting. The retention time (RT), monoisotopic exact mass, and molecular formula were all included in the database. The clustering of coeluting ions connected by charge-state, isotopic distribution, and/or the presence of various adducts and dimers in the analyzed samples was used for the feature-building process. The following adducts were selected for lipidomics analysis to detect coeluting adducts of the same feature: [M+H]+, [M+Na]+, [M+K]+, [M+NH4]+ and [M+C2H6N2+H]+ for positive ionization mode; [M-H]-, [M+Cl]-, [M+CH3COOH-H]-, and [M+CH3COONa-H]− for negative ionization. Common organic molecules (no halogens) for LC-ESI(+)-MS and common organic molecules for LC-ESI (-)-MS were used as the isotope grouping pattern, and the charge state was restricted to 1 - 2. We established the masses match tolerance at ± 20 ppm and ± 0.5 min for the RT. The RT score accounted for 100.00% of the total score. To complete the integration, we selected the "Agile 2” algorithm. The data format chromatogram employed was the centroid. The peak spectrum parameters were as follows: average scans > 10% of peak height, TOF spectra were excluded if above 20.0 % of saturation in the m/z ranges used, and empty spectrum will never return. The mass spectral data type was centroid. As a final post-processing filter, we only considered the features present in at least 50% of the samples in each time point.

**Data normalization and filtering**

Regarding lipidomics data, Data normalization was conducted utilizing the Kuligowski transformation 9 implemented in MATLAB (R2023a, MathWorks). This chemometric technique corrects batch effects post-acquisition, mainly targeting variability in QC samples and mitigating observed trends. Subsequently, lipid species were selected based on their coefficient of variation (CV) within the QC samples, applying a threshold of 30%.

**Multiplex immunoassays and ELISA**

A ProcartaPlex™ multiplex assay (Invitrogen™) was used to measure a range of anti-inflammatory/suppressor protein markers using a Luminex 200TM analyzer (Luminex Corporation, Austin, TX, United States): interleukin 8 (IL-8), IL-18, IL-1 receptor antagonist (IL-1RA), interferon-inducible protein 10 (IP-10), monocyte chemoattractant protein-1 (MCP-1), and endothelial dysfunction indicators like tumor necrosis factor receptor-1 (TNF-RI).

Additionally, the immuno-Oncology Checkpoint 14-Plex Human ProcartaPlex™ Panel 1 (InvitrogenTM) and 9-Plex Human ProcartaPlex™ Panel 3 were used to measure several plasma-soluble proteins following the manufacturer's guidelines using a Luminex 200TM analyzer (Luminex Corporation, Austin, TX, United States). Panel 1 included the following proteins: B and T lymphocyte attenuator (BTLA), cluster of differentiation 80 (CD80), CD152(CTLA4), indoleamine 2,3-dioxygenase (IDO), lymphocyte activation gene-3 (LAG-3), programmed cell death protein 1(PD-1), programmed death-ligand 1 (PD-L1), programmed death-ligand 2 (PD-L2), T-cell immunoglobulin and mucin-domain containing-3 (TIM-3), CD27, CD28, CD137(4-1BB), glucocorticoid-induced TNFR-related (GITR), and herpesvirus entry mediator (HVEM). Panel 3 measured B7-H6, CD276 (B7-H3), CD47 (integrin-associated protein (IAP)), CD48 (B-lymphocyte activation marker (BLAST-1)), CD134 (OX40), ICOS Ligand (B7-H2), T-cell immunoglobulin and mucin domain containing 4 (TIMD-4), S100A8/A9, and VISTA (B7-H5). Raw fluorescence intensity (FI) values were recorded in arbitrary units (a.u.).

# Supplementary Table 1. Biochemical markers of patients with HIV/HCV according to decompensated and compensated Advanced chronic liver disease.

|  | **All patients** | **dACLD** | **cACLD** | ***p*** |
| --- | --- | --- | --- | --- |
| **No.** | 58 | 10 (17.2%) | 48 (82.8%) |  |
| **Biochemical markers (mg/dL)** |  |  |  |  |
| Glucose | 101 (89–108) | 94 (89–123) | 101 (89–107) | 0.943 |
| Triglycerides (n = 57) | 118 (92–158) | 112 (90–138) | 118 (93–159) | 0.690 |
| Total cholesterol (n = 56) | 154 (134–173) | 157 (147–182) | 151 (133–173) | 0.563 |
| HDL cholesterol (n = 55) | 39 (30–56) | 39 (36–52) | 39 (30–56) | 0.965 |
| LDL cholesterol (n = 55) | 82 (64–104) | 94 (86–106) | 78 (59–104) | 0.169 |
| AST (IU/L) (n = 57) | 72 (46–113) | 52 (43–60) | 79 (52–123) | 0.018 |
| ALT (IU/L) | 64 (42–103) | 36 (28–62) | 72 (47–104) | 0.042 |
| Bilirubin | 0.9 (0.6–1.4) | 1.3 (0.8–1.8) | 0.8 (0.6–1.4) | 0.112 |
| Albumin (n = 57) | 4.1 (3.8–4.4) | 4.2 (4.0–4.4) | 4.1 (3.8–4.4) | 0.674 |

**Statistics**: The values are expressed as the median (interquartile range). P-values were calculated by the Mann-Whitney U test. **Abbreviations**: dACLD, decompensated advanced chronic liver disease; cACLD, compensated advanced chronic liver disease; HDL, high-density lipoprotein; LDL, low-density lipoprotein; AST, aspartate aminotransferase; ALT, alanine aminotransferase; IU, international units.

# Supplementary Table 2. Significant associations of lipids with decompensated advanced chronic liver disease (dACLD) in patients with HIV/HCV.

|  |  | **Un-adjusted** | | | **Adjusted** | |  |
| --- | --- | --- | --- | --- | --- | --- | --- |
| **Lipid** | **VIP** | **AMR (95%CI)** | ***p*-value** | ***q*-value** | **aAMR (95%CI)** | ***p*-value** | ***q*-value** |
| **LC-MS ESI+** |  |  |  |  |  |  |  |
| TG 42:1 | 2.45 | 0.97 (0.94 – 0.99) | 0.005 | 0.183 | 0.96 (0.94 – 0.99) | 0.005 | 0.148 |
| TG 44:2 | 1.84 | 0.97 (0.95 – 0.99) | 0.003 | 0.183 | 0.97 (0.95 – 0.99) | 0.003 | 0.148 |
| TG 45:2 | 1.46 | 0.98 (0.97 – 0.99) | 0.023 | 0.183 | 0.98 (0.97 – 0.99) | 0.023 | 0.168 |
| TG 53:2 | 1.49 | 0.98 (0.95 – 0.99) | 0.081 | 0.219 | 0.97 (0.93 – 0.99) | 0.009 | 0.148 |
| TG 58:7 | 2.20 | 0.95 (0.92 – 0.99) | 0.014 | 0.183 | 0.95 (0.93 – 0.98) | 0.007 | 0.148 |
| LPC 18:2/0:0 | 1.43 | 0.73 (0.57 – 0.93) | 0.016 | 0.183 | 0.76 (0.61 – 0.90) | 0.009 | 0.148 |
| LPE 20:4/0:0 | 1.39 | 0.74 (0.59 – 0.94) | 0.015 | 0.183 | 0.77 (0.64 – 0.91) | 0.018 | 0.163 |
| PC 38:3 | 1.61 | 0.79 (0.66 – 0.95) | 0.016 | 0.183 | 0.82 (0.68 – 0.94) | 0.021 | 0.168 |
| PC 16:0/18:2 | 1.77 | 0.70 (0.52 – 0.94) | 0.023 | 0.183 | 0.70 (0.51 – 0.90) | 0.017 | 0.163 |
| PC O-16:0/16:0 | 1.30 | 0.77 (0.61 – 0.96) | 0.023 | 0.183 | 0.75 (0.62 – 0.91) | 0.008 | 0.148 |
| PC O-16:1/18:2 | 1.19 | 0.73 (0.57 – 0.94) | 0.020 | 0.183 | 0.71 (0.57 – 0.88) | 0.011 | 0.155 |
| PC O-41:11 | 1.78 | 0.79 (0.64 – 0.97) | 0.026 | 0.188 | 0.79 (0.63 – 0.94) | 0.017 | 0.163 |
| PE 18:0/18:2 | 1.62 | 0.78 (0.66 – 0.92) | 0.005 | 0.183 | 0.79 (0.67 – 0.91) | 0.008 | 0.148 |
| PE 18:1/18:2 | 1.49 | 0.79 (0.65 – 0.95) | 0.019 | 0.183 | 0.79 (0.67 – 0.93) | 0.014 | 0.163 |
| PE 16:0/20:4 | 1.78 | 0.72 (0.54 – 0.94) | 0.022 | 0.183 | 0.71 (0.53 – 0.90) | 0.016 | 0.163 |
| PE O-18:2/18:2 | 1.31 | 0.91 (0.84 – 0.98) | 0.019 | 0.183 | 0.91 (0.85 – 0.99) | 0.010 | 0.155 |
| PE O-16:1/20:4 | 1.30 | 0.74 (0.59 – 0.94) | 0.018 | 0.183 | 0.71 (0.58 – 0.88) | 0.009 | 0.148 |
| PE O-20:1/18:3 | 1.58 | 0.84 (0.74 – 0.96) | 0.012 | 0.183 | 0.83 (0.73 – 0.94) | 0.004 | 0.148 |
| PS 21:2/20:4 | 1.89 | 0.82 (0.69 – 0.98) | 0.030 | 0.188 | 0.82 (0.68 – 0.97) | 0.022 | 0.168 |
| SM 39:0;O2 | 1.57 | 0.97 (0.94 –0.99) | 0.031 | 0.188 | 0.96 (0.94 – 0.99) | 0.014 | 0.163 |
| **LC-MS ESI-** |  |  |  |  |  |  |  |
| DG 36:4 | 2.41 | 0.94 (0.90 – 0.99) | 0.017 | 0.142 | 0.95 (0.92 – 0.98) | 0.017 | 0.100 |
| LPI 18:2/0:0 | 2.24 | 0.69 (0.51 – 0.91) | 0.013 | 0.142 | 0.69 (0.54 – 0.86) | 0.007 | 0.100 |
| PC 17:0/22:6 | 1.04 | 0.77 (0.61 – 0.96) | 0.024 | 0.142 | 0.77 (0.65 – 0.92) | 0.035 | 0.162 |
| PC O-18:1/18:2 | 1.28 | 0.75 (0.59 – 0.95) | 0.022 | 0.142 | 0.75 (0.60 – 0.92) | 0.012 | 0.100 |
| PI 16:0/18:2 | 1.42 | 0.75 (0.59 – 0.97) | 0.030 | 0.156 | 0.79 (0.67 – 0.95) | 0.030 | 0.156 |
| PA 34:2 | 1.34 | 0.86 (0.74 – 0.99) | 0.038 | 0.158 | 0.86 (0.76 – 0.98) | 0.016 | 0.100 |
| SM 16:1;O2/24:0 | 1.41 | 0.70 (0.52 – 0.93) | 0.017 | 0.142 | 0.70 (0.54 – 0.93) | 0.009 | 0.100 |
| FA 22:0 | 1.47 | 0.95 (0.91 – 0.99) | 0.012 | 0.142 | 0.95 (0.91 – 0.98) | 0.012 | 0.100 |

**Statistics**: Data were calculated by Generalized Linear Models (GLM) with a gamma distribution (log-link). Multivariable models were adjusted by age, gender, body mass index (BMI), HCV viral load and alcohol intake, which were previously selected by a stepwise method (forward) (see Results Section). The q-values represent p-values corrected for multiple testing using the False Discovery Rate (FDR; Benjamini and Hochberg procedure). **Abbreviations**: VIP, variable importance in projection; AMR, arithmetic mean ratio; aAMR, adjusted AMR; 95%CI. 95% of confidence interval; p, level of significance; q, corrected level of significance; DG, diglyceride; TG, triglyceride; LPC, lysophosphatidylcholine; LPE, lysophosphatidylethanolamine; LPI, lysophosphatidylinositol; PC, phosphatidylcholine; PE, phosphatidylethanolamine; PI, phosphatidylinositol; PS, phosphatidylserine; PA, phosphatidic acid; SM, sphingomyelin; FA, fatty acid.

# Supplementary Figure 1. Multivariate Orthogonal partial least squares discriminant analysis (OPLSDA) for decompensated and compensated advanced chronic liver disease (dACLD and cACLD) in patients with HIV/HCV.


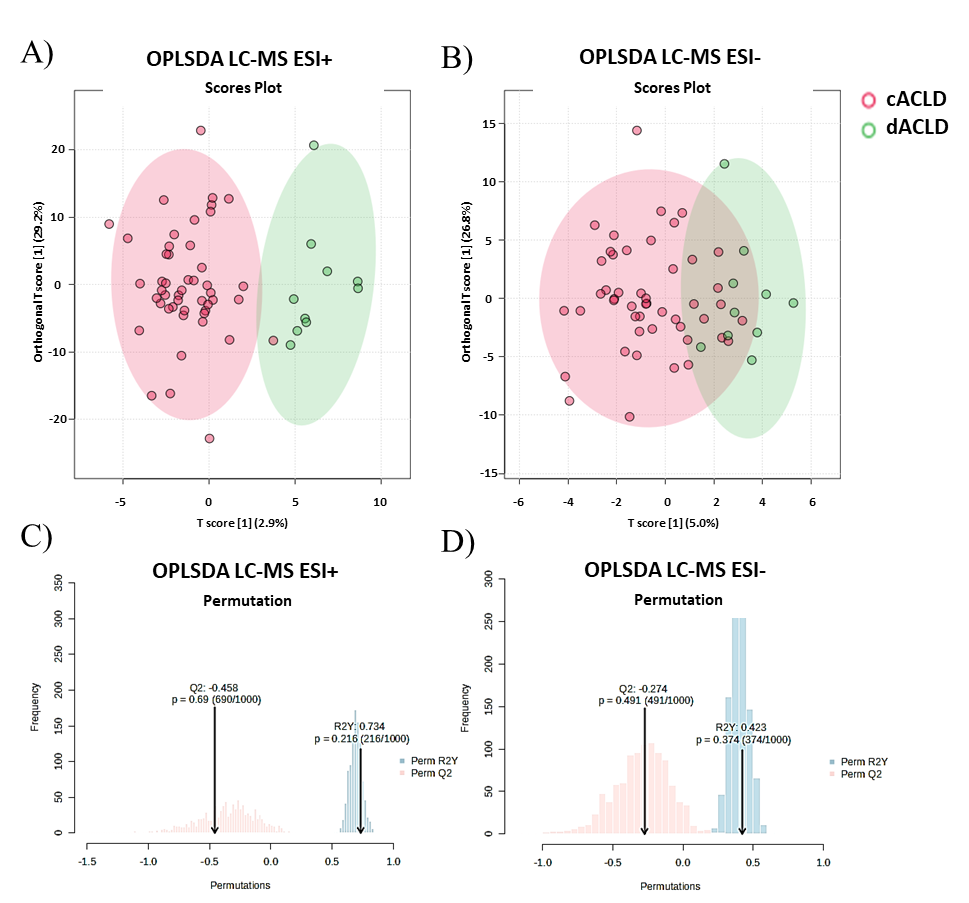


The figure displays the OPLS-DA and corresponding validation by permutation tests (1,000 permutations) for plasma lipidomic data in positive ionization mode (LC-MS ESI+) and negative ionization mode (LC-MS ESI-) from the study cohort (N=58). OPLS-DA scores plots for the **(A)** LC-MS ESI+ mode and **(B)** LC-MS ESI- mode. Permutation test validation plots (1,000 permutations) for the **(C)** LC-MS ESI+ OPLS-DA and **(D)** LC-MS ESI- OPLS-DA models.

**REFERENCES**

1. C. Gonzalez-Riano, A. Gradillas , C. Barbas. Exploiting the Formation of Adducts in Mobile Phases with Ammonium Fluoride for the Enhancement of Annotation in Liquid Chromatography High-Resolution Mass Spectrometry (LCHR-MS)-based Lipidomics. Journal of Chromatography Open. 2021:100018.

2. B. Fernández Requena, S. Nadeem, V. P. Reddy, et al. LiLA: lipid lung-based ATLAS built through a comprehensive workflow designed for an accurate lipid annotation. Communications Biology. 2024;7(1):45.

3. J. P. Koelmel, X. Li, S. M. Stow, et al. Lipid annotator: towards accurate annotation in non-targeted liquid chromatography high-resolution tandem mass spectrometry (LC-HRMS/MS) lipidomics using a rapid and user-friendly software. Metabolites. 2020;10(3):101.

4. H. Tsugawa, K. Ikeda, M. Takahashi, et al. MS-DIAL 4: accelerating lipidomics using an MS/MS, CCS, and retention time atlas. BioRxiv. 2020:2020.2002. 2011.944900.

5. Z. Ni, G. Angelidou, M. Lange, R. Hoffmann , M. Fedorova. LipidHunter identifies phospholipids by high-throughput processing of LC-MS and shotgun lipidomics datasets. Analytical Chemistry. 2017;89(17):8800-8807.

6. M. I. Alcoriza-Balaguer, J. C. García-Cañaveras, A. n. López, et al. LipidMS: an R package for lipid annotation in untargeted liquid chromatography-data independent acquisition-mass spectrometry lipidomics. Analytical chemistry. 2018;91(1):836-845.

7. M. I. Alcoriza-Balaguer, J. C. García-Cañaveras, F. J. Ripoll-Esteve, M. Roca , A. Lahoz. LipidMS 3.0: an R-package and a web-based tool for LC-MS/MS data processing and lipid annotation. Bioinformatics. 2022;38(20):4826-4828.

8. A. Gil-De-La-Fuente, J. Godzien, S. Saugar, et al. CEU Mass Mediator 3.0: A Metabolite Annotation Tool. Journal of Proteome Research. 2019;18(2):797-802.

9. J. Kuligowski, Á. Sánchez-Illana, D. Sanjuán-Herráez, M. Vento , G. Quintás. Intra-batch effect correction in liquid chromatography-mass spectrometry using quality control samples and support vector regression (QC-SVRC). Analyst. 2015;140(22):7810-7817.
